# Unusual giant low-grade appendiceal mucinous neoplasm: A case report and literature review

**DOI:** 10.1097/MD.0000000000042828

**Published:** 2025-06-06

**Authors:** Xuhui Ma, Wei Dong, Qing Yang, Jie Yu, Shunchang Zhou, Yuxu Zhong, Haibo Chu

**Affiliations:** a Department of General Surgery, Jiaozhou Branch of Shanghai East Hospital, Tongji University, Qingdao, Shandong, P.R. China; b Department of Pathology, Jiaozhou Branch of Shanghai East Hospital, Tongji University, Qingdao, Shandong, P.R. China; c State Key Laboratory of Toxicology and Medical Countermeasures, Beijing Institute of Toxicology and Pharmacology, Beijing, P.R. China.

**Keywords:** diagnosis, laparoscopic surgery, low-grade appendiceal mucinous neoplasm

## Abstract

**Rationale:**

Low-grade appendiceal mucinous neoplasm (LAMN) is a rare subtype of appendiceal pathology characterized by epithelial hyperplasia, cellular atypia, and mucinous accumulation within the appendiceal lumen, leading to obstructive expansion of the organ. Representing a mere 0.2% to 0.3% of all appendectomies and approximately 0.5% of gastrointestinal tumors, LAMN poses diagnostic challenges in surgical practice.

**Patient concerns:**

We conducted a retrospective analysis of a patient with the appendiceal neoplasm. A 39-year-old female presented to Jiaozhou Hospital, East Hospital Affiliated to Tongji University on July 9, 2022, complaining of a nine-day history of right lower abdominal pain, distension, and nausea. Physical examination revealed a palpable measuring 15 × 6 cm in the right lower quadrant, accompanied by tenderness, rebound tenderness, and muscular guarding.

**Diagnoses:**

Histopathological examination confirmed the diagnosis of a LAMN without evidence of lymphovascular invasion, serosal, or mesenteric infiltration.

**Interventions:**

After antiinflammatory therapy and comprehensive assessment, the neoplasm was excised through three-dimensional laparoscopic surgery.

**Outcomes:**

No recurrence was observed during an 32-month postoperative follow-up period.

**Lessons:**

Despite its rarity, LAMN warrants clinical attention due to its nonspecific symptoms. Computed tomography scans significantly improve preoperative diagnostic accuracy, with pathological diagnosis serving as the gold standard. Surgical intervention is the preferred treatment option, albeit controversies persist regarding surgical extent and the utilization of preoperative and postoperative chemotherapy.

## 1. Introduction

Low-grade appendiceal mucinous neoplasm (LAMN) represents a well-differentiated, slow-growing tumor.^[1,2]^ Despite its clinical rarity, LAMN accounts for approximately 0.2% to 0.3% of appendectomy specimens and 0.5% to 1% of gastrointestinal neoplasms. It predominately affects individuals in the 50 to 60 age group, with a higher incidence observed in females.^[3,4]^ The 2019 World Health Organization (WHO) classification of digestive system neoplasms includes proliferative and serrated polyps, LAMN, high-grade appendiceal mucinous neoplasm (HAMN), and appendiceal mucinous adenocarcinoma.^[5]^

HAMN is characterized by appendicular mucinous tumors displaying LAMN-like structures, noninvasive invasion, and high-grade cellular atypia.^[6]^ In HAMN, the appendix often presents with mucous cysts or is enveloped in mucinous masses within the ileocecal region. Portions of the appendiceal lumen may exhibit cystic dilation, gradual thinning of the tube wall, and accumulation of thick mucus, potentially leading to appendiceal tube wall rupture and mucus overflow into the serous membrane and mesenteries.^[7–9]^

Pseudomyxoma peritonei was first defined by Werth in 1884 as a clinical syndrome characterized by tumorous cells implantation in the abdominal cavity, often manifesting as mucous ascites, peritoneal implantation, and involvement of the omentum and ovaries.^[6,10]^ Its incidence rate is approximately 2 per 1,000,000 individuals,^[11]^ with recent years witnessing a rising trend, reaching approximately 3 to 4 per 1,000,000 individuals per year.^[12,13]^

LAMN exhibits benign cytological characteristics but harbors invasive biological potential, with the potential to progress into pseudomyxoma peritonei.^[14]^ Clinical manifestations of LAMN are nonspecific and often mimic those of acute or chronic appendicitis, typically presenting with palpable masses and right lower abdominal pain as principal symptoms. This similarity often results in high rates of misdiagnosis and missed diagnoses, posing a significant diagnostic challenge to surgeons. Herein, we present a case of LAMN from our institution alongside a comprehensive review of the literature.

## 2. Case presentation

**Figure 1.**
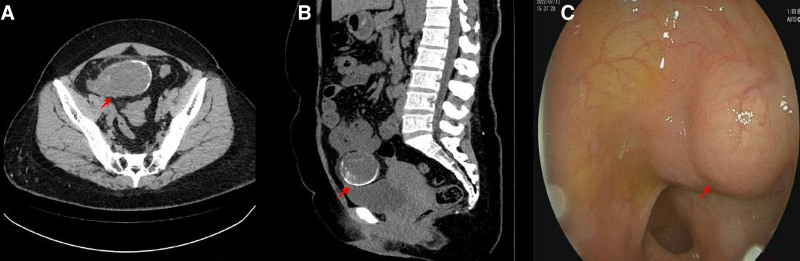
Contrast-enhanced computed tomography (CT) and endoscopic images in low-grade appendiceal mucinous neoplasm (LAMN). (A) Axial view of the enhanced CT image displays an eggplant-like swelling in the appendix with heterogeneous low density and patchy enhancement within the lumen. (B) Sagittal CT image depicts cystic dilation of the appendix featuring a widened root, enhanced wall, and calcifications reminiscent of eggshells. (C) Colonoscopy image reveals normal mucosa, a spherical impression in the submucosal layer, displacement of the appendiceal orifice, and soft tissue intercepted by biopsy forceps (indicated by arrows).

**Figure 2.**
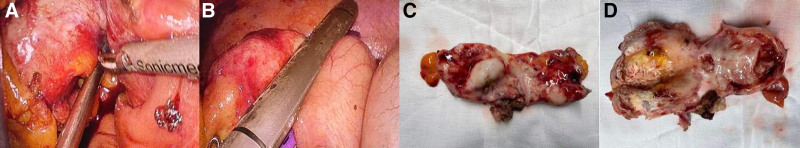
Intraoperative images and corresponding specimens in LAMN. (A) Appendiceal separation via an ultrasonic scalpel under three-dimensional laparoscopy. (B) Appendiceal root division employing a cutting and sealing device. (C) Enlarged appendix revealing irregular wall thickening and inflammatory edema. (D) Appendiceal wall exhibiting uneven thickening with translucent modifications, and the lumen filled with copious amounts of gelatinous mucus.

**Figure 3.**
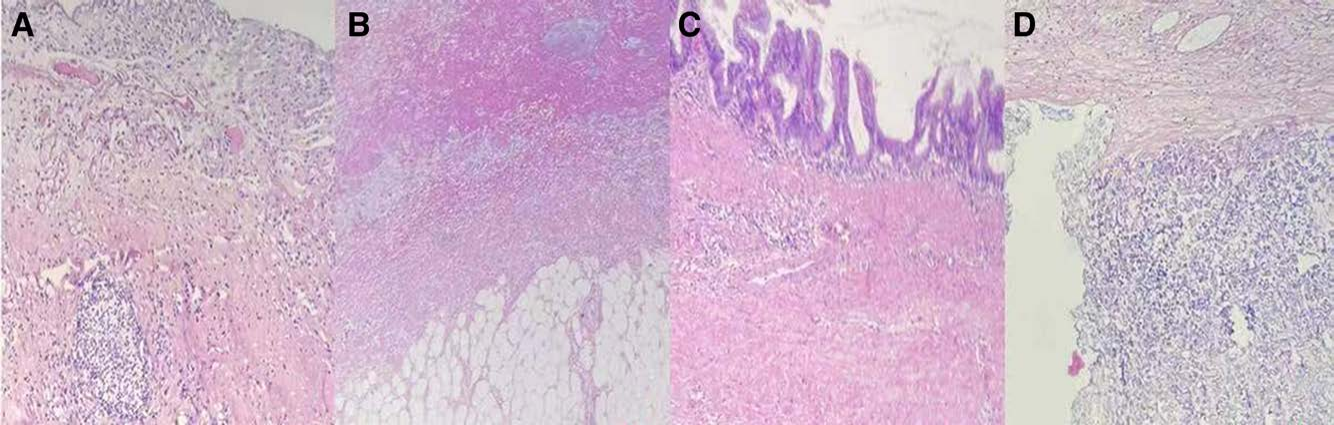
Hematoxylin and eosin (HE) stained histological slides in LAMN. (A) Increased vascularity in the subserosal layer of the appendix. (B) Absence of cellular mucin and translucent alterations in the appendiceal wall. (C) Wavy configuration of the mucinous epithelium, concomitant with basement membrane loss and submucosal fibrosis. (D) Vanishing of the mucosal muscle layer, indicative of “pushing invasion” (HE × 100, bars = 100 μm).

**Figure 4.**
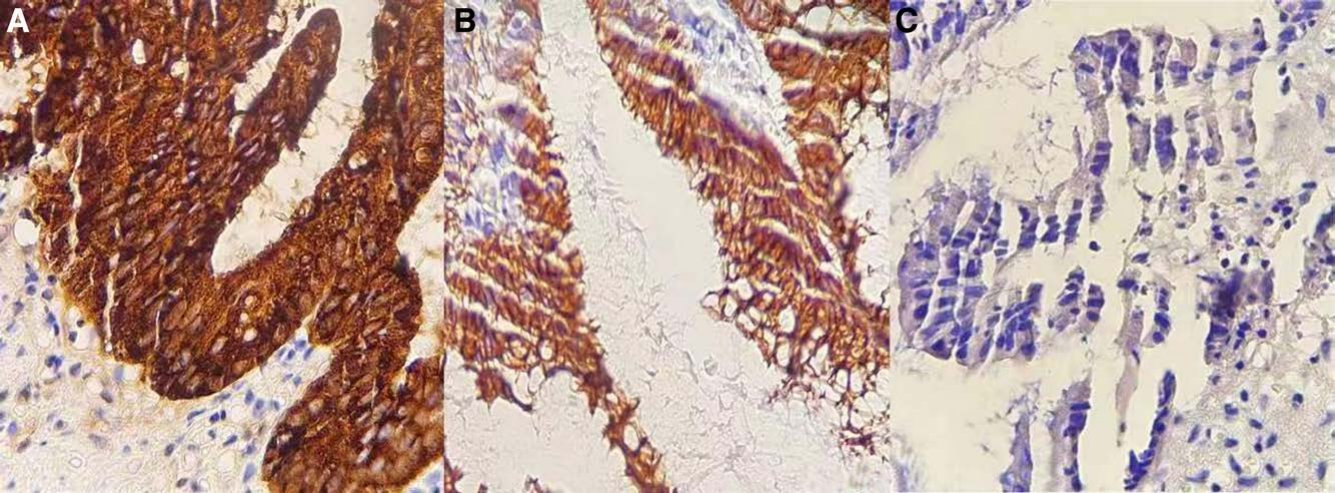
Immunohistochemistry (IHC) images in LAMN. (A) High positive expression of MUC2 in the mucinous epithelium of the appendix. (B) Positive expression of CK20 in the mucinous epithelium of the appendix. (C) Absence of CK7 expression in the mucinous epithelium of the appendix (MUC2, CK20, and CK7 immunolabeling, × 400 magnification, Bars = 25 µm). CK7 = cytokeratin 7, CK20 = cytokeratin 20, MUC2 = mucin 2.

A 39-year-old female presented to our hospital on July 9, 2022, complaining of a nine-day history of right lower abdominal pain, distension, and nausea. Physical examination revealed a palpable measuring 15 × 6 cm in the right lower quadrant, accompanied by tenderness, rebound tenderness, and muscular guarding. Bowel sounds were within normal limits. Laboratory investigations revealed a white blood cell count of 9.25 × 10^9^/L, predominantly neutrophils (76.6%), hemoglobin concentration of 103 g/L, and elevated tumor markers, including carcino-embryonic antigen (CEA) (65 ng/mL), carbohydrate antigen-125 (CA125) (85 U/mL), and carbohydrate antigen-199 (CA199) (329 U/mL). Enhanced computed tomography (CT) imaging of the abdomen and pelvis revealed a small cluster in the right lower quadrant with slight enhancement and homogenous thickening of the intestinal wall. The appendix appeared elongated and cystic, measuring approximately 13 cm × 5 cm^2^, with punctate areas of high density and wall calcifications, alongside enhancement of the appendiceal wall. A colonoscopy revealed a smooth, spherical lesion protruding into the cecum from the appendiceal orifice. Biopsy forceps encountered soft tissue (Fig. 1A–C), consistent with an appendiceal neoplasm. The patient received preoperative antiinflammatory therapy and underwent further diagnostic evaluation. On July 17, a 3D laparoscopic appendectomy was performed. Intraoperative findings included minimal pale yellow peritoneal fluid and an inflammatory mass in the right lower quadrant due to adhesions between the terminal ileum, pelvic cavity, and abdominal wall. The appendiceal root was thickened, measuring 2.0 cm in diameter, with the body exhibiting neoplastic dilation, approximately 13 × 6 × 5 cm. No peritoneal implants were noted. Adhesions were meticulously dissected, and the appendiceal mesentery was divided using an ultrasonic scalpel. The tumor was completely excised, and the appendix was removed at its base with a cutting and closing device (Fig. 2A–D). Histopathological examination confirmed the diagnosis of a LAMN without evidence of lymphovascular invasion, serosal, or mesenteric infiltration (Fig. 3A–D). Immunohistochemical staining analysis revealed positive expression of mucin 2 and cytokeratin 20 (CK20) in the mucinous epithelium of the appendix, with the absence of cytokeratin 7 (CK7) expression (Fig. 4A–C). At the 18-month postoperative follow-up, abdominal ultrasound, CT scan, and serum tumor marker assays revealed no evidence of tumor recurrence.

All patient-identifying information has been removed. The study was approved by Jiaozhou Branch of Shanghai East Hospital, Tongji University Ethics Committee. The patient provided written consent for both treatment and publication of this report. The reporting of this study conforms to CARE guidelines.^[15]^

## 3. Discussion

The term “LAMN” was introduced by Misdraji^[16]^ in 2010 and subsequently adopted by WHO. LAMN is characterized by its relatively indolent nature, often undergoing prolonged growth periods before the onset of discernable clinical symptoms.^[17]^ Due to its nonspecific clinical presentation, diagnosis of LAMN can be delayed. Early stages of the disease typically manifest as appendiceal enlargement due to mucus accumulation within the appendiceal cavity, resulting in right lower abdominal pain resembling appendicitis. Advanced stages may present with abdominal distension due to peritoneal mucous implantation and ascites.^[18]^

In a single cohort study by Bell et al^[19]^, which involved 117 patients with LAMN, it was observed that the disease predominantly affects women with a male-to-female ratio of 1:2. The average age of patients was 60 years, with the majority being of Caucasian descent (91%). Clinical symptoms were present in 97% of patients, with abdominal pain (56%) and a diagnosis of appendicitis (75%) being common, Similarly, Gündoğar et al^[20]^, following the WHO 2010 guidelines, identified 19 LAMN cases, with symptoms including right lower abdominal pain (27%), palpable abdominal masses (16%), weight loss (10%), and changes in bowel habits (5%), and a male-to-female ratio of 2:7. Xiao et al^[21]^ reported a higher female prevalence (88%) in their review of 51 LAMN cases, with symptoms ranging from abdominal pain (31%) and distension (22%) to abdominal masses (16%), rectal bleeding (2%), and associated symptoms (27%). Preoperative diagnoses varied and included appendiceal cysts (39%), uncertain pelvic masses (18%), adnexal masses (18%), and ovarian cancer (20%).

LAMN often presents as mild appendiceal dilation, typically self-resolving. Moreover, increased intracavitary pressure due to mucus accumulation induces epithelial cell atrophy, which is responsible for mucus secretion, thereby reducing mucus production and limiting appendix expansion.^[4]^ However, complications, such as appendiceal perforation or rupture due to mucinous cysts can occur. Benign or malignant mucinous cysts, along with nonneoplastic mucinous cysts like proliferative and retention cysts—the latter frequently due to appendiceal fecalith or extrinsic compressive masses^[22]^—must be considered in clinical settings. Previous research^[2,23]^ suggested that 23% to 50% of asymptomatic LAMN cases could be highly suspected based on preoperative imaging, endoscopic examination, and intraoperative findings, with 60% to 65% of patients exhibiting elevated CA199, CA125, and CEA levels. Advanced medical imaging has significantly improved the preoperative diagnostic rate of LAMN. The clinical presentation of LAMN varies widely, from asymptomatic cases to acute or chronic abdominal pain, often mimicking acute appendicitis, posing diagnostic challenges for surgeons.^[24]^

In our case, the patient presented with right lower abdominal pain and signs of local peritonitis. During the operation, hyperemia and edema of the tumor cyst wall were observed, along with extensive inflammatory adhesions to the surrounding bowel, greater omentum, and abdominal wall, consistent with the typical clinical features of LAMN. Complications associated with LAMN include intestinal obstruction, intestinal perforation, cyst rupture, pseudomyxoma peritonei, colonic intussusception, and extraintestinal fistula.^[23,25,26]^ Rare associations with appendiceal endometriosis, rectal cancer, and HAMN have also been reported.^[27–29]^ In clinical practice, differential diagnoses of LAMN should consider ovarian cancer, appendiceal diverticulum, and low-grade appendiceal mucinous neoplasm.^[30–32]^

Typical ultrasound findings of LAMN include an irregular cystic structure with linear anechoic fluid within the appendiceal region, accompanied by punctate blood flow signals. Other signs have been reported as appendiceal wall calcification, exhibiting an “onion skin” appearance, and an appendiceal diameter exceeding 1.5 cm.^[33]^ Atypical ultrasound manifestations also include mucinous cysts, intestinal obstructions of the small bowel, intestinal intussusception, appendicitis, appendiceal rupture, and HAMN.^[22]^ Typical CT attributes of LAMN often demonstrate a mass lesion in the right lower abdomen, and appendiceal dilation, appearing as a round, oval, or elongated cystic mass resembling an elongated eggplant. Furthermore, the lesion may present as unilocular or multilocular with septations, with its size frequently correlating to disease progression. The cyst wall also displays varying thicknesses and exhibits multiple punctate, arc-shaped, or eggshell-like calcifications. Additionally, the lesion’s boundaries may range from well-defined to indistinct. A significant amount of gelatinous mucus, demonstrating a uniform density and a CT value below 20 HU, fills the cyst.^[34]^

In certain cases, the appendix may exhibit partial dilation, taking on a pear-shaped or chicken-leg-like appearance. Atypical features observed through CT scans for LAMN include irregular thickening of the cyst wall, adjacent soft tissue changes, and the presence of bubbles or air-fluid levels inside the cystic cavity.^[35]^ Soon et al^[4]^ reported that an appendiceal diameter below 0.6 cm on CT could hint at LAMN, while a diameter exceeding 2 cm should raise suspicion for LAMN in instances of acute appendicitis. Similarly, Xiao et al^[21]^ proposes that CT findings such as calcification of the appendiceal wall and an appendix diameter exceeding 1.3 cm could serve as diagnostic markers for LAMN, indicating CT’s superiority over ultrasound for LAMN diagnosis. Magnetic resonance imaging (MRI) is primarily used to differentiate right-sided appendiceal lesions from other mucinous cystic lesions of appendiceal and ovarian origin. However, MRI is also effective in assessing extraluminal mucus, appendiceal rupture, LAMN, and appendiceal adenocarcinoma. Mucinous cysts typically appear as high signal intensity on T2-weighted MRI images, with T1-weighted intensity varying based on mucin content, often appearing as low signal intensity. Extraluminal mucin may also exhibit high signal intensity on T2-weighted images. Importantly, MRI surpasses CT in detecting peritoneal implants. For pseudomyxoma peritonei, diffusion-weighted imaging and delayed enhancement imaging improve the clarity of peritoneum and viscera, aiding in preoperative evaluation and postoperative assessment for metastatic spread and determination of suitability for cytoreductive surgery (CRS) and hyperthermic intraperitoneal chemotherapy (HIPEC). MRI provides crucial information for these considerations.^[34]^ However, CT is superior to MRI in depicting cyst wall calcifications.^[36]^

In this case, the ultrasound of the right lower abdomen revealed an enlarged appendix with a thin and complete cystic wall, accompanied by calcification and an “onion skin” appearance. Enhanced abdominal CT showed an eggplant-like enlargement of the appendix, featuring cystic dilation with a widened base, heterogeneous low density, patchy enhancement within the lumen, enhanced wall, and calcifications resembling eggshells. Colonoscopy revealed normal mucosa, a spherical impression in the submucosal layer, displacement of the appendiceal orifice, and soft tissue sampled by biopsy forceps. These ultrasonography and imaging findings were consistent with LAMN characteristics. The imaging differential diagnosis of LAMN includes simple appendicitis, periappendicular abscess, mucinous cystadenoma, cystadenocarcinoma of the appendix, and cyst or cystadenoma originating from the right adnexa.

According to the classification by the Peritoneal Surface Oncology Group International in 2016,^[37]^ LAMN falls within a category characterized by penetration beyond the mucosal layer into the appendix wall. These tumors demonstrate a push-invasive growth pattern and show low-grade cellular dysplasia. Macroscopically, LAMN presents as a significantly enlarged appendix with a substantial volume of mucinous content within the lumen. The appendiceal wall typically displays either transparent or fibrotic modifications.^[24]^ Histologically, LAMN primarily exhibits low-grade cellular dysplasia and dominant proliferative growth of mucinous epithelial cells, arranged in either a villous or flat pattern. Microscopic examination often reveals the absence of the mucosal layer, fibrosis in the submucosal region, invasive proliferation into adjacent tissues, lack of cellular mucin in the appendiceal wall, wave-like or flat epithelial growth patterns, with occasional instances of appendiceal rupture or mucin production.^[38]^ LAMN demonstrates unique biological characteristics, including a tendency toward minimal lymph node engagement and hematogenous dissemination.^[35]^

Bell et al^[19]^ conducted a comprehensive pathological analysis of 117 LAMN cases, revealing flat mucinous epithelium in 38% of cases and a villous or wave-like configuration in 62%. Additionally, they noted epithelial exposure and appendiceal dilation in 79% of cases; tiny calcifications within the lumen and wall in 63%, and transparent or fibrotic changes in the appendiceal wall in 82%. Additionally, goblet cell proliferation was evident in 27% of cases, along with the absence of the mucosal layer in 99%, submucosal fibrosis in 78%, and a “pushing invasion” characteristic in 32%. Furthermore, mucinous cells were identified within the appendiceal lumen in 27% of cases. Gündoğar et al^[20]^ performed a pathological examination on 19 LAMN cases, identifying low-grade cellular dysplasia in 58% of cases, mucosal layer disappearance in 47%, and submucosal fibrosis in 58%. Notably, none of the cases included appendix perforation. However, wave-like or flat epithelial growth was observed in 26% of cases, lymph node atrophy in 42%, the “pushing invasion” feature in 5% and transparent or fibrotic changes in the mucosal layer in 63%. Conversely, studies by Soucisse et al^[39]^ and Reiter et al^[40]^ suggested that low-grade cellular dysplasia, “pushing invasion,” and the absence of mucinous cells infiltrating or penetrating the appendiceal wall serve as distinctive pathological indicators of LAMN. Akay et al^[41]^ noted that patients with LAMN exhibiting mucosal layer disappearance had a 50% incidence of malignancy, while those lacking mucosal layer disappearance but displaying submucosal fibrosis (72.1%) had a 25% malignant transformation rate. Additionally, 20.9% of LAMN cases were associated with appendiceal diverticula. In cases of diverticulum rupture, mucin can spread into the abdominal cavity, leading to pseudomyxoma peritonei. The histological characteristics of “pushing invasion” are described as extensive epithelial cell expansion into the appendiceal wall, leading to the formation of diverticulum-like structures. This process results in circumferential thinning of the appendiceal wall, accompanied by transparent and densely fibrotic submucosa. Notably, this process lacks cellular mucin exerting pressure on the appendiceal wall.^[42]^

Immunohistochemistry plays a significant role in LAMN diagnosis. Schmoeckel et al^[43]^ reported robust nuclear positivity for special AT-rich sequence-binding protein 2, while recombinant caudal type homeobox 2 and CK20 were positively expressed. Conversely, CK7 demonstrated a negative expression. In an investigation by Aldaoud et al.^[44]^ Immunohistochemical analysis on 8 LAMN cases and one adenocarcinoma case demonstrated that all cases (100%) exhibited positive caudal type homeobox 2 expression, while paired box 8 was negatively expressed in all cases (100%). Furthermore, CK7 was positively expressed in 3 cases (33.3%), and negatively expressed in 6 cases (66.7%). CK20 was positively expressed in 8 cases (88.9%), and special AT-rich sequence-binding protein 2 in 7 cases (77.8%). A retrospective study by Gok et al^[45]^ performed immunohistochemical analysis on 18 LAMN cases, indicating positive CK20 expression in all cases (100%), while negative CK67 expression was observed in 71% of cases. Moreover, mucin 5 subtype AC and deleted in pancreatic cancer 4 showed positive expression in 86% and 100% of cases, respectively.

In this case, the enlarged appendix exhibited irregular wall thickening and inflammatory edema, with the appendiceal wall showing nonuniform thickening and translucent modifications. Excessive gelatinous mucus was observed in the lumen, along with increased vascularity in the subserosal layer. Microscopic examination revealed features consistent with LAMN, including the absence of cellular mucin and translucent alterations in the appendiceal wall, wavy configuration of the mucinous epithelium, features concomitant with basement membrane loss and submucosal fibrosis, diminishing mucosal muscle layer, and characteristics indicative of “pushing invasion.” Immunohistochemical staining confirmed positive expressions of mucin 2 and CK20, and the absence of CK7 expression in the mucinous epithelium of the appendix. Overall, our results suggested a tumor of epithelial tissue origin with no epithelial cell proliferation. Pathological differential diagnoses for LAMN encompass appendiceal diverticulum, adenoma (tubular, villous, or tubulovillous), serrated polyp, HAMN, appendiceal goblet cell carcinoma, mucinous carcinoma with signet ring cells, and signet ring cell carcinoma with adenocarcinoma.^[20,46,47]^

Consensus regarding the optimal surgical strategy for managing LAMN has been reached, yet controversy surrounds the surgical management of cases with potential mucinous spillage. A retrospective analysis of 277 LAMN cases from multiple Japanese centers revealed differing viewpoints. Some researchers advocate for CRS in cases where LAMN extends beyond the appendiceal cavity to minimize the risk of pseudomyxoma peritonei. Conversely, others highlight the low incidence of lymph node metastasis in LAMN, suggesting postoperative HIPEC without advocating for extensive colon resection. Additionally, some suggest appendectomy with mesenteric lymph node dissection for LAMN, while for G2 or G3 patients, extended colon resection with lymph node dissection is recommended.^[37]^ Young et al^[48]^ argue that simple appendectomy is sufficient for LAMN, without the need for extensive resection. Sueda et al^[49]^ in their analysis of 138 LAMN cases, found that appendectomy alone was performed in 75 cases (54.3%), cecum resection in 26 cases (18.8%), right hemicolectomy in 37 cases (26.8%), CRS in 7 cases (5.1%), and HIPEC in 3 cases (3.3%). Similarly, Xiao et al^[21]^ suggest that simple appendectomy is adequate for LAMN, but if the tumor involves the appendiceal serosa, right hemicolectomy or ileocecal resection may be necessary. Legué et al^[23]^ advocate for appendectomy followed by observation for patients without extra-appendiceal lesions while advising against right hemicolectomy. For patients with positive surgical margins, cecal resection is recommended.

The 2020 Chicago Consensus on peritoneal malignancies provides several recommendations for managing LAMN. The guidelines recommend appendectomy, with cases having positive proximal surgical margins necessitating appendiceal cuff resection or partial cecal resection. The guidelines also emphasize limiting the use of ileocecal resection or right hemicolectomy.^[41]^ In a retrospective evaluation of 116 LAMN cases conducted by Pai et al^[50]^, it was reported that 81% of patients underwent appendectomy, whereas 19% underwent a combination of appendectomy, right hemicolectomy, and terminal ileum resection. Yang et al^[51]^ argue that following an appendectomy for LAMN, right hemicolectomy should be considered for cases with positive surgical margins, lymph node involvement, or appendiceal perforation. They also advocate for prophylactic HIPEC. McDonald et al^[52]^ and Enomoto et al^[53]^ recommend CRS combined with HIPEC for asymptomatic patients with perforations, leading to recurrence rates of 0% and 13.3%, respectively. Singhal et al^[54]^ analyzed 41 LAMN cases, with appendectomies performed in 34 cases, right hemicolectomy in 2 cases, and gynecological surgery in 5 cases.

Koizumi et al^[55]^ advocate laparoscopic resection as the preferred surgical method for LAMN, emphasizing intraoperative measures to prevent tumor rupture and PMP development. Amano et al^[56]^ detailed 4 LAMN cases, with 3 undergoing laparoscopic appendectomy and one laparoscopic ileocecal resection with lymph node dissection, all resulting in zero postsurgical recurrences. Bonomi et al^[57]^ suggest laparoscopic appendectomy for LAMN cases without serosal involvement, while open surgery is recommended for cases with appendiceal rupture or peritoneal implants. Indications for right hemicolectomy proposed by Shaib et al^[18]^ include poorly differentiated cells, increased mitotic activity, appendiceal serosal involvement, lymph node metastasis, tumor size exceeding 2 cm, and perforation of high-grade, poorly differentiated tumor perforation. In this case, appendectomy was performed using 3D laparoscopy. The procedure showcased technical advantages in precise adhesion separation and tumor excision. Importantly, the tumor was found intact without mucous lesions or intraperitoneal implantation in the appendiceal serosa, thus, a simple appendectomy was deemed appropriate.

In cases of LAMN with extracolonic dissemination, the overall survival rates at 3, 5, 7, and 10 years were documented as 100%, 86%, 60%, and 45%, respectively, when appendectomy or right hemicolectomy were utilized as treatment modalities. Adjuvant chemotherapy employing 5-FU is recommended for high-grade undifferentiated neoplasms, particularly those with lymph node metastasis or tumor perforation, while it is discouraged for low-grade, well-differentiated neoplasms.^[27]^ Literature suggests categorizing LAMN into 2 epithelial types: noncellular mucinous and cellular mucinous, each potentially impacting progression-free survival and overall survival. The cellular mucinous type exhibits a 7.5-times greater risk of recurrence compared to the non-cellular mucinous type.^[58]^ For LAMN cases achieving complete resection, prognosis generally tends to be favorable, with no significant concerns. However, the necessity for follow-up remains debated. Longitudinal follow-up revealed that 95.2% of patients did not experience recurrence within 5 years. Annual abdominal CT scans and serum tumor marker tests (CEA, CA199, and CA125) are recommended for 5 to 10 years.^[38]^ A prognostic study by Sueda et al^[49]^ on 138 LAMN cases, with an average follow-up duration of 61.3 months, showed that 12 cases (8.7%) developed pseudomyxoma peritonei, with an overall survival rate of 94.9%. A comparative study by Shin et al^[59]^ involving 26 cases of appendiceal mucinous adenoma and 20 cases of LAMN demonstrated 5-year overall survival rates of 95.5% and 93.8%, respectively, with progression-free survival rates at 95.2% and 95%, respectively. Among the 18 LAMN cases, 2 (11.1%) reported recurrence. Kitai et al^[60]^ analyzed 250 LAMN cases with follow-up durations ranging from 0.1 to 135 months, documenting 5 cases of recurrence (2%) and a 5-year overall survival rate of 94.5%. Recent studies have revealed that approximately 20% of patients with LAMN develop pseudomyxoma peritonei around 12.4 months post-appendectomy, advocating for regular follow-ups, including CT scans and serum tumor marker evaluations every 4 to 6 months.^[40]^ In a study by Huang et al^[61]^ the 10 -year survival rate for LAMN was noted as 32%. However, with CRS + HIPEC surgical intervention, the 5-year survival rate could increase to as high as 80%. At the 18-month postoperative follow-up, abdominal ultrasound, CT scan, and serum tumor marker assays revealed no evidence of tumor recurrence.

In conclusion, LAMN represents a clinically rare condition with well-defined diagnostic criteria. Its clinical presentation is often nonspecific, and the use of imaging, endoscopy, and serum tumor marker tests has proven beneficial for diagnosis. Surgical intervention remains the primary therapeutic approach, with CRS combined with HIPEC showing significant efficacy in preventing recurrence and progression to pseudomyxoma peritonei. However, areas of disagreement persist regarding surgical techniques and prognostic outcomes, highlighting the need for further investigation through comprehensive, multi-center, longitudinal studies.^[62]^

## Acknowledgments

We would like to thank Prof Lu Bing, Department of Anorectal Surgery in Shanghai East Hospital, Tongji University for our guidance and help to us.

## Author contributions

**Conceptualization:** Haibo Chu, Wei Dong, Yuxu Zhong.

**Data curation:** Xuhui Ma, Shunchang Zhou.

**Formal analysis:** Qing Yang.

**Funding acquisition:** Yuxu Zhong.

**Investigation:** Haibo Chu, Xuhui Ma, Wei Dong.

**Methodology:** Xuhui Ma, Qing Yang, Jie Yu, Shunchang Zhou.

**Resources:** Jie Yu, Shunchang Zhou.

**Software:** Jie Yu, Shunchang Zhou.

**Writing – review & editing:** Haibo Chu, Yuxu Zhong.

**Writing – original draft:** Xuhui Ma, Wei Dong.
